# Genetic resources and genes/QTLs for gram pod borer (*Helicoverpa armigera* Hübner) resistance in chickpea from the Western Himalayas

**DOI:** 10.1002/tpg2.20483

**Published:** 2024-07-04

**Authors:** Sheikh Aafreen Rehman, Shaheen Gul, M. Parthiban, Ishita Isha, M. S. Sai Reddy, Annapurna Chitikineni, Mahendar Thudi, R. Varma Penmetsa, Rajeev Kumar Varshney, Reyazul Rouf Mir

**Affiliations:** ^1^ Division of Entomology, Faculty of Agriculture (FoA) SKUAST‐Kashmir Kashmir India; ^2^ Department of Agricultural Biotechnology and Molecular Biology Dr. Rajendra Prasad Central Agricultural University (RPCAU) Pusa India; ^3^ Department of Entomology Dr. Rajendra Prasad Central Agricultural University (RPCAU) Pusa India; ^4^ Centre for Crop and Food Innovation WA State Agricultural Biotechnology Centre Murdoch University Murdoch Western Australia Australia; ^5^ College of Agriculture, Family Sciences and Technology Fort Valley State University Fort Valley Georgia USA; ^6^ Department of Plant Science University of California, Davis (UC‐Davis) Davis California USA; ^7^ Division of Genetics & Plant Breeding Faculty of Agriculture (FoA) SKUAST‐Kashmir Kashmir India

## Abstract

*Helicoverpa armigera* (also known as gram pod borer) is a serious threat to chickpea production in the world. A set of 173 chickpea genotypes were evaluated for *H. armigera* resistance, including mean larval population (MLP), percentage pod damage (PPD), and pest resistance (PR) for 2 consecutive years (year 2020 and 2021). The same core set was also genotyped with 50K *Axiom CicerSNP Array*. The trait data and 50,000 single nucleotide polymorphism genotypic data were used together to work out marker–trait associations (MTAs) using different genome‐wide association studies models. For MLP, a total of 53 MTAs were identified, including 25 MTAs in year 2020 and 28 MTAs in year 2021. A set of three MTAs was found common in both environments. For PPD, two MTAs in year 2020 and five MTAs in year 2021 were identified. A set of two MTAs were common in both environments. Similarly, for PR, only two MTAs common in both environments were identified. Interestingly, a common MTA (Affx_123255526) on chromosome 2 (Ca2) was found to be associated with all the three component traits (MLP, PPD, and PR) of pod borer resistance in chickpea. Further, we report key genes that encode SCAMPs (that facilitates the secretion of defense‐related molecules), quinone oxidoreductase (enables the production of reactive oxygen species that promotes diapause of gram pod borer), and NB‐LRR proteins that have been implicated in plant defense against *H. armigera*. The resistant chickpea genotypes, MTAs, and key genes reported in the present study may prove useful in the future for developing pod borer–resistant chickpea varieties.

## Core Ideas

- A core set of 173 chickpea genotypes was evaluated for *Helicoverpa armigera* resistance for 2 years. Pod borer–resistant genotypes were identified.
- Genotyping of core set with *Axiom CicerSNP Array* has ∼50,000 single nucleotide polymorphisms.
- A total of 53 significant marker–trait associations/genes/quantitative trait loci conferring resistance to *H. armigera* were identified.
- Candidate gene analysis identified key genes responsible for plant defense against *H. armigera* (mean larval population) in chickpea.

**Abbreviations**
GLM: general linear model
GWAS: genome‐wide association studies
MLM: mixed linear model
MLMM: multi‐locus mixed model
MTA: marker–trait association
QTL: quantitative trait locus
SNP: single nucleotide polymorphism

## 1 INTRODUCTION

Globally, chickpea (*Cicer arietinum* L.) is cultivated in more than 50 countries on 13.72 million ha with an annual production of 14.25 million metric tons (Food and Agriculture Organization [FAO], 2020) in the arid and semi‐arid regions of the world. Although India ranks first by contributing to 65% of global production, by 2050 we need to achieve an average productivity of 15–17 q/ha (Dixit et al., 2019) for attaining sustainability and nutritional demands. Several biotic and abiotic stresses have been keeping the productivity below 1 ton per ha. Among abiotic stresses, drought, heat, and salinity are major, often leading to about 70% yield losses (Jha et al., 2022; Varshney et al., 2019). Nevertheless, the availability and phosphorous‐use efficiency also play an important role in attaining production demands (Thudi et al., 2021).

Fungal pathogens like *Fusarium oxysporum ciceris* in warmer regions and *Ascochyta rabeii* in cooler regions have been hampering chickpea production. Several national and international efforts enabled the identification of the genomic regions responsible for the resistance to these key diseases (Garg et al., 2018). In addition, molecular breeding varieties have been developed and released for cultivation in the case of Fusarium wilt (Laxuman et al., 2024; Mannur et al., 2019). Apart from fungal diseases, insect damage is more pronounced than the other stresses. The most important pests of chickpea are cutworms and termites during seedling stages in certain areas, while the gram pod borer, *Helicoverpa armigera*, appears in vast numbers during the vegetative and pod formation stages of chickpea (Veer et al., 2020). *H. armigera* is a serious, polyphagous pest commonly called the American bollworm, gram pod borer, or fruit borer in India. Under normal weather conditions in India, up to 60% yield losses were reported (Bhatt & Patel, 2001), while 50%–100% losses were reported under favorable climatic conditions, particularly within the states where frequent rains and cloudy weather prevail during the crop season (Jaba et al., 2017; Kumar & Durairaj, 2012; Saminathan et al., 2003).

This pest infests 225 plant species belonging to 46 families in India, including both widely grown and economically important crops such as cotton, maize, tobacco, pigeon pea, chickpea, and tomato (Reddy, Agnihotri, Divija, et al., 2022). In addition, gram pod borers are cosmopolitan in the tropics and subtropics and are found in several crops in Africa, Australia, Southeast Asia, New Zealand, and Southern Europe. It is also referred to as tomato fruit borer and cotton bollworm. On the basis of the crop it feeds.

Due to fast generation turnover, high reproductive rates, and wide genetic diversity, certain organisms can cause severe damage to crops, invade new hosts, and exhibit intraspecies variability, including adaptation to different environments and climates (Deepa & Srivastava, 2011; Reddy, Agnihotri, Jaiswal, et al., 2022). It is estimated that *H. armigera* alone is responsible for losses of over Rs. 35,000 million annually in India despite heavy pesticide inputs. Although it attacks chickpea throughout crop growth, the damage caused during flowering and pod formation stages leads to substantial yield losses. Insecticides are costly, and their indiscriminate use has induced insect resistance to most insecticides in *H. armigera* (Honnakerappa & Udikeri, 2018) and led to environmental pollution (Dey, 2016). Hence, the identification of new sources of resistance through screening in hotspot regions as well as the development of resistance to *Helicoverpa* through modern breeding approaches are potential alternatives for attaining sustainability.

Nevertheless, few efforts were made to understand the resistance trait studies in chickpea against gram pod borer, and it was reported that a quantitative trait locus (QTL) cluster on CaLG03 holds great potential to improve *H. armigera* resistance component traits in chickpea (Barmukh et al., 2021). Molecular markers provide valuable tools for entomologists, ecologists, and plant breeders for the characterization of crop germplasm, the identification of insect‐resistant germplasm, and gene discovery for insect resistance (Yencho et al., 2000). Association mapping or genome‐wide association studies (GWAS) have recently become popular for identifying marker–trait associations (MTAs) in plant populations (Gangurdae et al., 2022; Thudi et al., 2014, 2023). Compared to linkage mapping, genome‐wide association studies have several advantages. It requires less time and resources, provides higher mapping resolution, and evaluates a wide range of alleles rapidly (Zhu et al., 2008).

In the Western Himalayan regions of Kashmir, primarily focused on mountain agriculture, chickpea is grown in both summer and winter seasons. During harsh winter, the crops face huge cold stress up to minus (−5 to −7°C), followed by early spring temperature rises. *Helicoverpa* emerges from overwinter and causes infestation in chickpea. As chickpea is well adopted for the summer season, we were successful in developing and releasing varieties like Shalimar chickpea‐02, the first summer chickpea variety for the Kashmir valley. Despite this achievement, *Helicoverpa* damage is also posing a threat to summer cultivation (May–August). In order to understand the major constraints to chickpea production in Western Himalayan regions, we constituted a diverse core set of chickpea genotypes (see Fayaz et al., 2019, 2022; Mir et al., 2021). Using this diverse chickpea core set, the current study aimed to identify chickpea genotypes exhibiting resistance to *Helicoverpa* infestation and also unravel the molecular markers associated to this resistance into pod borer resistance through GWAS.

**Core Ideas**

- A core set of 173 chickpea genotypes was evaluated for *Helicoverpa armigera* resistance for 2 years. Pod borer–resistant genotypes were identified.
- Genotyping of core set with *Axiom CicerSNP Array* has ∼50,000 single nucleotide polymorphisms.
- A total of 53 significant marker–trait associations/genes/quantitative trait loci conferring resistance to *H. armigera* were identified.
- Candidate gene analysis identified key genes responsible for plant defense against *H. armigera* (mean larval population) in chickpea.

## 2 MATERIALS AND METHODS

### 2.1 Plant material

In the present study, a core set of 173 diverse chickpea genotypes, including desi (brown, black, and green seeded), kabuli (cream and beige seeded), and pea‐shaped, was evaluated for their relative resistance against *H. armigera*. All the genotypes belong to the cultivated gene pool and no wild relatives (e.g., *Cicer reticulatum*, *Cicer echinospermum*, etc.) are involved in the study. The genotypes were procured from various national/international institutes, namely, the International Crops Research Institute for the Semi‐Arid Tropics (ICRISAT), Hyderabad, the Rafi Ahmad Kidwai (RAK) College of Agriculture, Rajmata Vijayaraje Schindia Krishi Vishwa Vidyalaya (RVSKV), Sehore, Madhya Pradesh, the Indian Council of Agricultural Research (ICAR)‐Indian Institute of Pulse Research (IIPR), Kanpur, and the ICAR‐National Bureau of Plant Genetic Resources (NBPGR), New Delhi. More details about the planting material are available elsewhere (see Fayaz et al., 2021, 2019, 2022; Mir et al., 2021). Furthermore, we have selected one resistant check Y10 and two susceptible checks Y13 and Y5 to compare with the other genotypes for recording data on pod borer infestation.

### 2.2 Experimental design and data recording

The core set of 173 genotypes was sown in an augmented block design (ABD) in the research field of the Division of Genetics and Plant Breeding, Faculty of Agriculture (FoA), SKUAST‐K, Wadura Campus, Sopore, Jammu and Kashmir, India (34°17′ N latitude, 74° 33′ E longitude, and at an altitude of 1594 m above sea level). The experiments were conducted on clay loam soil, rich in organic matter, phosphorus, and potassium, with an acidic pH. Seeds were hand‐drilled and sown at a depth of approximately 3 cm. All the genotypes were sown in 4‐m‐long rows with inter‐ and intra‐row spacing of 45 and 10 cm. Three check genotypes, Y5 (susceptible check), Y10 (resistant check), and Y13 (susceptible check), were sown after every 10 rows/genotypes. The checks were selected based on their response to pod borer infestation in our earlier studies and not on the basis of yield which we believe is directly correlated to pod borer damage. Standard agronomic packages were adopted during both crop seasons.

The weather during the crop growth period from May to August in Kashmir is considered a typical summer weather. For instance, in year 2020–2021, the average high/day temperature was 28.5°C, while the average low/night temperature recorded was 13.8°C. Similarly, the average rainfall recorded during this period was 2.8 mm, and the maximum relative humidity was found to be 75.3%. The minimum relative humidity recorded was 49.1% during the crop growth period. In the subsequent year (2021–2022) (May–August), the average high/day temperatures were 27.6°C, while the average low/night temperature recorded was 15.04°C. Similarly, the average rainfall recorded during this period was 3.6 mm, and the maximum relative humidity was found to be 77.71%. The minimum relative humidity recorded was 55.64% during the crop growth period.

With the aim of assessing the extent of natural variation and resistance to pod borer damage, no insecticide was sprayed on the crop. For recording the data, the Y13 genotype was used as a susceptible check in which a single larva of *H. armigera* has been found to cause damage for 25–30 pods, and infestation of this susceptible check genotype was considered as starting point for recording trait data on other genotypes. We selected five plants randomly for each genotype for recording the trait data on each component trait of pod borer resistance. For instance, the mean larval population (MLP) was recorded at weekly intervals from pest appearance to maturity of the crop for five random plants/genotype.

The percentage pod damage (PPD) was calculated from the five randomly selected plants by using the following formula:

$$\begin{array}{ccc} \mathrm{Percent}\;\mathrm{pod}\;\mathrm{damage} & = & \mathrm{Number}\;\mathrm{of}\;\mathrm{damaged}\;\mathrm{pods}\\ & & /\mathrm{Total}\;\mathrm{no}.\;\mathrm{of}\;\mathrm{pods}\times 100 \end{array}$$

For calculating the pest resistance (1–8 scale; Table S1), the percentage of pod damage in the test genotypes was compared with that of the check varieties (Reddy & Agnihotri, 2018). The test genotypes were then graded using the following formula for pest resistance:

$$\begin{array}{ccc} & & \mathrm{Pest}\;\mathrm{resistance}\left(\%\right)\\ & & =\frac{\mathrm{Percent}\;\mathrm{PD}\;\mathrm{in}\;\mathrm{check}\;\mathrm{cultivar}-\mathrm{Percent}\;\mathrm{PD}\;\mathrm{in}\;\mathrm{test}\;\mathrm{cultivar}}{\mathrm{Percent}\;\mathrm{PD}\;\mathrm{in}\;\mathrm{check}\;\mathrm{cultivar}}\\ & & \;\times \;100 \end{array}$$

where PD is pod damage.

All the trait data that have been recorded for all the three component traits of pod borer resistance have been used for GWAS in combination with genotypic data.

#### 2.2.1 Morphological trait data recorded on chickpea genotypes with different pod borer pest resistance

A set of 21 chickpea genotypes, including susceptible check Y13, were used during the present study. The genotypes belonging to each category/scale of pod borer pest resistance were selected from the 2‐year phenotyping result and further characterized for leaf trichome density and pod wall thickness. The genotypes including resistant genotypes to highly susceptible genotypes (Table 1). Trichome density was measured in accordance with Jackai and Oghiakhe (1989). The leaves were cut with scissor and placed in acetic acid and alcohol (2:1) in glass vials for 24 h to clear the chlorophyll (Maiti & Bidinger, 1979). The presence of trichomes was recorded in minimum of five leaves from each accession. The leaf sections were mounted on a glass slide and examined under a microscope. Pod wall thickness was measured using the vernier calipers for five random pods per genotype. Three measurements in each pod were taken and averaged to compute pod wall thickness, which was represented in mm.

**TABLE 1:** Mean number of trichomes per leaf and pod wall thickness in selected chickpea genotypes selected across the pest‐resistant scales.

Genotype	Pest resistance scale	Mean number of trichomes/leaf	Mean pod wall thickness (mm)	Category
NK 7	3	298 ± 25.345	1.28 ± 0.108	Resistant
Y 14	3	286.67 ± 24.383	1.19 ± 0.100	Resistant
NK 3	4	274.83 ± 23.373	1.22 ± 0.106	Moderately resistant
NK 31	4	241.83 ± 20.567	0.94 ± 0.079	Moderately resistant
NK 70	4	249.17 ± 21.192	0.96 ± 0.079	Moderately resistant
NK 55	4	263.5 ± 22.409	0.98 ± 0.085	Moderately resistant
CC 92	5	191.67 ± 16.303	0.82 ± 0.070	Intermediate
CC 154	5	196 ± 16.670	0.84 ± 0.071	Intermediate
H 18	5	226.83 ± 19.291	0.93 ± 0.079	Intermediate
CC 35	5	170.33 ± 14.485	0.73 ± 0.062	Intermediate
CC 131	5	187.83 ± 15.974	0.79 ± 0.067	Intermediate
CC 29	6	175.67 ± 14.942	0.75 ± 0.064	Moderately susceptible
CC 16	6	160 ± 13.608	0.61 ± 0.053	Moderately susceptible
CC 19	6	166.17 ± 14.133	0.64 ± 0.053	Moderately susceptible
CC 34	6	150 ± 12.757	0.57 ± 0.047	Moderately susceptible
CC 38	6	139.67 ± 11.880	0.52 ± 0.044	Moderately susceptible
CC 27	7	126.83 ± 10.786	0.50 ± 0.041	Susceptible
Y 8	8	117.67 ± 10.009	0.49 ± 0.041	Highly susceptible
CC 111	8	73.33 ± 6.235	0.29 ± 0.026	Highly susceptible
CC 120	8	50.83 ± 4.322	0.24 ± 0.021	Highly susceptible
Y 13 (susceptible check)	8	100.17 ± 8.520	0.35 ± 0.032	Highly susceptible
CD	–	47.585	0.194	–
SE (m)	–	16.615	0.068	–
CV (%)	–	15.709	15.78	–

Abbreviations: CD, critical difference; CV, coefficient of variation; SE, standard error.

### 2.3 Genotyping using *Axiom CicerSNP Array*

Genomic DNA was isolated from 15‐day‐old seedlings following the high‐throughput mini DNA isolation protocol suggested by Cuc et al. (2008). We genotyped the 173 core germplasm lines using an *Axiom CicerSNP* Array that contains 50,590 high‐quality single nucleotide polymorphisms (SNPs) that are distributed all over the chickpea genome (Roorkiwal et al., 2018). For genotyping, 100 ng of DNA was randomly fragmented, purified, and hybridized to *Axiom CicerSNP Array* as per Roorkiwal et al. (2018). Further, stringent conditions were followed, nonspecific random ligations were washed‐off, and the array was stained, imaged, and processed using the GeneTitan Multi‐Channel instrument to generate the data. The SNP scoring was done as per Roorkiwal et al. (2018).

### 2.4 Genome‐wide association analysis

For the identification of MTAs, a total of 7277 polymorphic SNPs obtained from the genotyping of a core set of 173 chickpea genotypes as well as the phenotyping data recorded on these genotypes were used. GWAS was performed using genotypic data and trait data recorded on a core set of 173 chickpea genotypes, including three check genotypes, to identify genes/QTLs/markers associated with *H. armigera* resistance. This association analysis was performed using three different statistical models, namely, general linear model (GLM), mixed linear model (MLM), and multi‐locus mixed model (MLMM), of the software program TASSEL, and the results were summarized in the form of Manhattan plots, QQ plots, and tables. In Manhattan plots, the *p*‐values for each SNP are plotted against its genomic location. The *p*‐values are transformed as −10 log (*p*) so that their higher values mean stronger significance. In QQ plots, a comparison is made between the two distributions, where one is the expected distribution and the other is the realized distribution. In GWAS, the QQ plot usually shows the realized and sorted *p*‐values for all SNPs against their expected values (Fayaz et al., 2022).

### 2.5 Mining of candidate genes and haplotypes

The physical position of 17 unique significant markers associated with *H. armigera* resistance, identified using three different statistical models, namely, GLM, MLM, and MLMM during the present study, were used for mining candidate genes in these regions on the chickpea reference genome assembly‐v1 (Varshney et al., 2013). The gene ontology (GO) terms associated with the genes were searched for GO terms using BLAST2GO software (Conesa et al., 2005).

## 3 RESULTS AND DISCUSSION

Chickpea is a major pulse crop grown all over the world, including India. It is rich in carbohydrates, micronutrients, and proteins together, constituting approximately 80% of the total dry seed mass in comparison with other pulses (Chibber et al., 2010). Being rich in proteins, it is susceptible to insect pests. During recent years, a decline in production from 16.94 to 15.87 million metric tons in 2021 has been reported (Statista, 2023). The decline in production may be due to various reasons including biotic and abiotic stresses. The pod borer infestation has been found to cause substantial yield losses. For instance, 10%–60% yield loss was reported under normal weather conditions, while as under favorable conditions in states with frequent rains and cloudy weather, the yield loss ranges from 50% to 100% (Saminathan et al., 2003; Sarwar et al., 2009). Polyphagous nature of pod borer makes the pest control using insecticides not much effective, and integrated pest management has been advocated to minimize the losses due to gram pod borer (El Fakhouri et al., 2022). Although baculoviruses are used as biopesticides, reduced efficacy due to the inactivation of the baculoviruses on the leaf surface of chickpea and other legumes has been reported to be ineffective (Aminu et al., 2023). Further, antifeedant and larvicidal properties of some natural extract were also studied to control gram pod borer (Boulamtat et al., 2020). Understanding the genetic variability and capturing the allelic history will enable to come up with strategies to develop superior lines with enhanced resistance to *Helicoverpa*. Globally, several efforts were made to understand and catalogue the existing variation for resistance to *Helicoverpa* (Dias et al., 1983; El Fakhouri et al., 2022; Shafique et al., 2009; Sharma et al., 2005). We report our efforts to understand the extent of diversity in germplasm accessions to gram pod borer as well as the allelic history of key loci associated with resistance and putative candidate genes that can be used for enhancing resistance to gram pod borer.

### 3.1 Germplasm evaluation under field conditions

#### 3.1.1 Large variation for larval population

Evaluation of 173 chickpea genotypes during 2020 and under natural field conditions for *H. armigera* resistance revealed highly significant differences in the levels of resistance between different accessions. For instance, the MLP among different genotypes ranged between 0.20 and 5.45 per plant. The lowest larval population was found in genotypes NK 7, NK 63, and NK 48 (0.20 per plant). The highest MLP was found in genotype H3 (5.45 per plant) and was found to be most susceptible among all the genotypes. During 2021, the MLP ranged between 0.22 and 5.47 per plant. The lowest larval population was found in genotype NK 48 (0.22 per plant), and this genotype was found to be superior to all other genotypes. The highest MLP was found in genotype H3 (5.47 per plant) and was found to be most susceptible among all the genotypes (Table S2). Similarly, the larval population varied from 0.33 to 5.67 per plant (Veer et al., 2020). Our findings align with previous reports of Nadeem et al. (2010), Rajput et al. (2003), Rashid et al. (2003), and Reddy and Agnihotri (2018), who documented the differential response of chickpea germplasm to *Helicoverpa* infestation with varying level of resistance. Thus, the genotype NK 48 supported the smallest number of populations during both years and hence can be considered the best candidate genotype for breeding pod borer–resistant chickpea varieties. In addition, the genotype can also be used in genomics, transcriptomics, and metabolomics studies to understand the mechanism of pod borer resistance in chickpea.

#### 3.1.2 Wide variation for percentage pod damage

During 2020, the PPD among different genotypes was found to vary between 2.50% and 73.39%. The lowest PPD was found in genotypes NK7 and NK 63, and the highest was found in genotype H3. During 2021, the PPD was found to vary between 2.54% and 74.78%. The lowest percentage damage during 2021 was found in genotype “NK7,” and the highest damage was found in genotype H3. During both years, the lowest PPD was found in the genotype “NK 7” (Table S2). During 2021, nine genotypes were found to be resistant, 14 genotypes were moderately resistant, 22 genotypes were intermediate, 22 genotypes were moderately susceptible, 18 genotypes were susceptible, and 85 genotypes were highly susceptible (Table S2). Our studies revealed much variation among different genotypes in response to *H. armigera*. This may be due to differences in morphological and biochemical characteristics of resistance among various genotypes. The genotypes with less larval population and pod damage will prove very useful in chickpea breeding programs. As many pesticides are being used for the control of *H. armigera* in chickpea, the use of genotypes with inbuilt resistance against pod borers in this study will help to reduce pesticide usage, slow the development of resistance, and can also be used for further resistance breeding programs. In another study, evaluation of perennial wild relatives of chickpea indicated that percentage damage rating was <2.0 in case of accessions belonging to *Cicer microphyllum*, *Cicer canariensis*, and *Cicer macracanthum*, while it was 4.0 in *Cicer judaicum* and 8.5–9.0 in the case of cultivated chickpeas (Sharma et al., 2006).

#### 3.1.3 Morphological characteristics contributing to pod bore resistance

The average trichome density quantified on leaves of a set of 20 test genotypes from each category of pod borer pest resistance scale varied between 50.83 and 298 per leaf. The maximum number of trichome was found in resistant genotypes NK7, Y14, and NK3 with 298 ± 25.34, 286.67 ± 24.3, and 273.83 ± 23 trichomes/leaf, respectively (Table 1). On the other hand, lowest number of trichomes were recorded in susceptible genotypes CC120, CC111, and Y8 with 50.83 ± 4.3, 73.33 ± 6.2, and 117.67 ± 10 trichomes/leaf (Table 1). Similarly, pod wall thickness revealed that mean pod wall thickness on different genotypes varied between 0.24 and 1.28 mm. The highest pod wall thickness was recorded in resistant genotypes NK 7, NK 3, and Y 14, and lowest pod wall thickness was recorded in susceptible genotypes CC120 and CC111 (Table 1).

The trichomes found on the leaf surfaces of plants are known to play a crucial role in plant defense against various pests and pathogens. The results indicate that genotypes with higher trichome densities, such as NK 7, Y 14, and NK 3, demonstrated enhanced resistance to pod borer pests. These observations align with some previous studies, where similar findings, reinforcing the significance of trichomes in conferring resistance against pod borer pests in chickpea, have been reported (Girija et al., 2008; Shahzad et al., 2005; Sharma et al., 2009).

Similarly, pod wall thickness is also considered as another potential factor contributing to resistance against pod borer pests. Our results showed that genotypes with thicker pod walls, such as NK7, NK3, and Y7, exhibited greater resistance to pod borer compared to genotypes with thinner pod walls. These finding are consistent with earlier finding of Girija et al. (2008) where thicker pod walls have been found to act as a physical barrier, making it more difficult for pod borer pests to penetrate and feed on developing seeds. By selecting genotypes with higher trichome densities and thicker pod walls, breeders can potentially enhance the resistance of chickpea varieties against pod borer pests, thereby reducing yield losses and dependency on chemical pesticides. The promising genotypes with high number of trichomes and thicker pod wall will prove useful in chickpea breeding programs aimed at developing pod borer–resistant varieties.

### 3.2 Genome‐wide association studies for *H. armigera* resistance

#### 3.2.1 GWAS signals for larval populations

**FIGURE 1:**
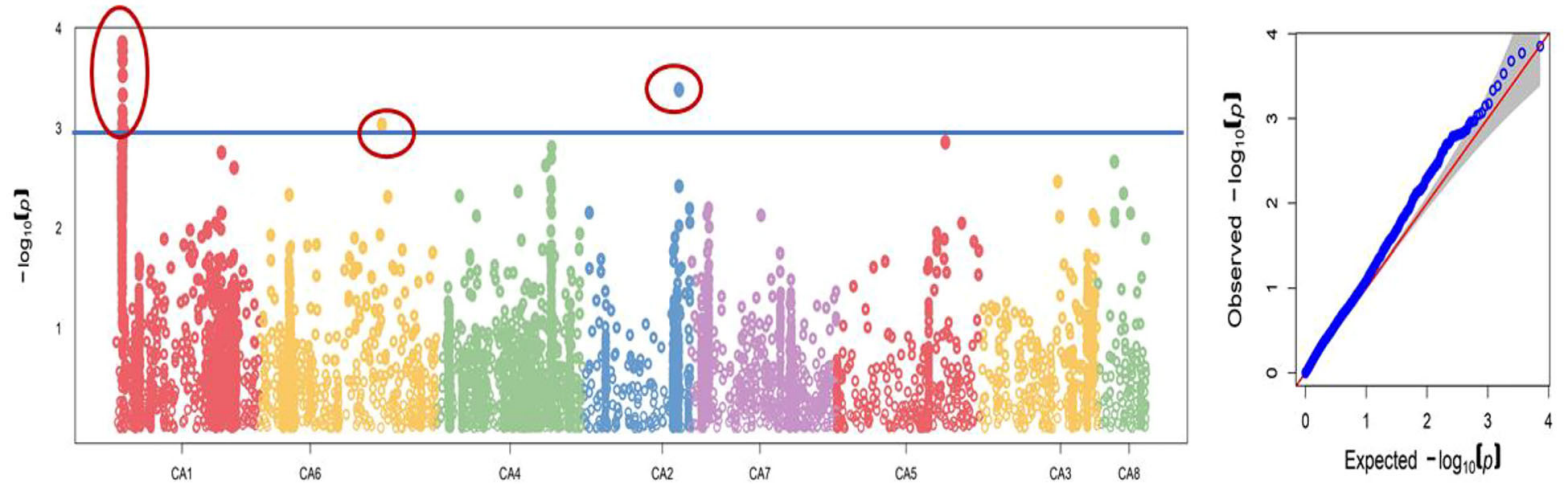
Manhattan and QQ plots showing candidate single nucleotide polymorphism (SNP) marker loci and *p*‐values from GWAS for *Helicoverpa armigera* resistance based on larval population of year 2020. (The blue line on Manhattan plot is the fixed significance threshold of −log_10_ (*p* value) at 3).

**FIGURE 2:**
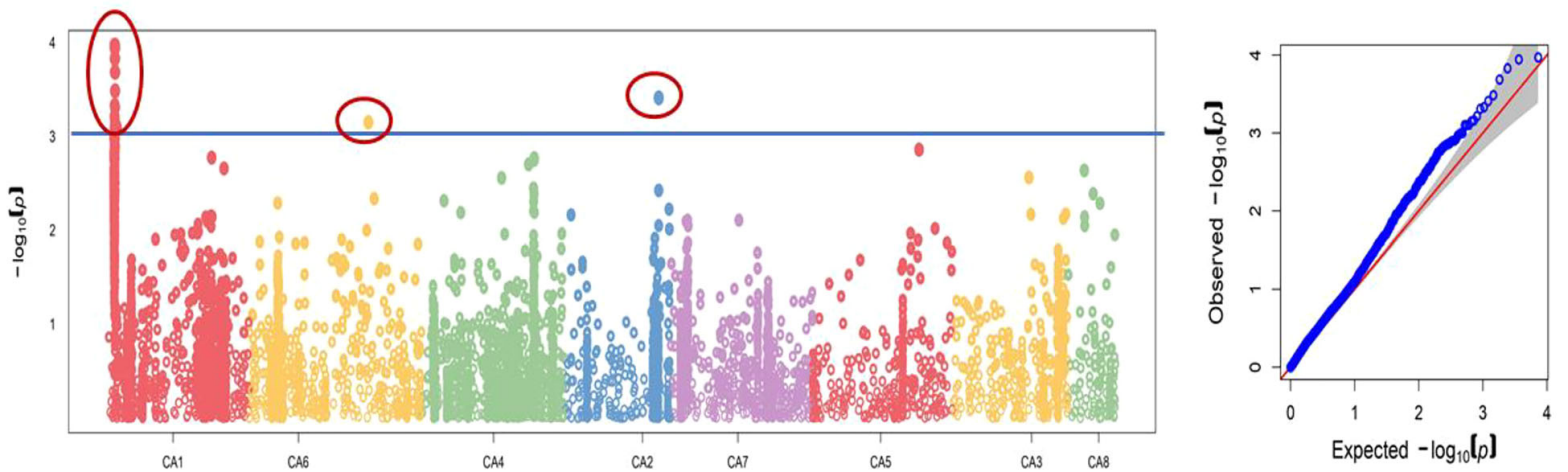
Manhattan and QQ plots showing candidate single nucleotide polymorphism (SNP) marker loci and *p*‐values from GWAS for *Helicoverpa armigera* resistance based on larval population of year 2021. (The blue line on Manhattan plot is the fixed significance threshold of −log_10_ (*p* value) at 3).

Using GLM, we identified 11 and 14 significant MTAs for larval populations during 2020 and 2021, respectively, on Ca1, Ca2, and Ca6 (Table 2; Figure 1). The use of MLM provided five significant MTAs, each based on the phenotyping data recorded in year 2020 as well as year 2021. Further, using MLMM, a set of nine significant MTAs each was identified based on phenotyping data recorded in year 2020 and 2021, respectively, and these SNPs are present on Ca1, Ca2, and Ca6; some of the MTAs overlapped between models (Table 2). A few representative Manhattan and QQ plots showing MTAs detected using different models are shown in Figures 1 and 2. In earlier studies using biparental mapping population derived from cross ICC 4958 × PI489777 and *Axiom CicerSNP Array*, nine main‐effect QTLs and 955 epistatic QTLs were reported. These QTLs were located on Ca1, Ca3, Ca4, and Ca6 linkage groups (Barmukh et al., 2021). Furthermore, we identified three (Affx_123255526, Affx_123241676, and Affx_123248246) MTAs on Ca1 and Ca2 in both the years (years 2020 and 2021) and therefore declaed as stable MTAs (Table 2; Figures 1 and 2). These stable MTAs will prove useful in future chickpea breeding programs.

**TABLE 2:** Details of marker–trait associations (MTAs) identified for *Helicoverpa armigera* larval population using different GWAS (genome‐wide association studies) models in different years.

SNP	Chromosome	Position	*p* value
**Significant markers depicted by GLM showing association with *Helicoverpa armigera* larval population (2020)**			
Affx_123241676	Ca1	2,026,559	0.00014
Affx_123248246	Ca1	2,081,797	0.000169
Affx_123249471	Ca1	2,083,627	0.00021
Affx_123286825	Ca1	2,123,224	0.000296
Affx_123255526	Ca2	32,467,962	0.000412
Affx_123249101	Ca1	2,139,423	0.000465
Affx_123245103	Ca1	2,011,348	0.00067
Affx_123286346	Ca1	2,132,822	0.000711
Affx_123253041	Ca1	2,102,245	0.000851
Affx_123287807	Ca1	1,975,258	0.000892
Affx_123277156	Ca6	41,065,459	0.000921
**Significant markers depicted by MLM showing association with *Helicoverpa armigera* larval population (2020)**			
Affx_123255526	Ca2	32,467,962	0.000468
Affx_123266834	Ca6	43,044,918	0.000584
Affx_123241676	Ca1	2,026,559	0.000666
Affx_123242495	Ca1	35,358,570	0.000829
Affx_123248246	Ca1	2,081,797	0.000871
**Significant markers depicted by MLMM showing association with *Helicoverpa armigera* larval population (2020)**			
Affx_123255526	Ca2	32,467,962	0.000224
Affx_123266834	Ca6	43,044,918	0.000295
Affx_123241676	Ca1	2,026,559	0.000346
Affx_123242495	Ca1	35,358,570	0.000452
Affx_123248246	Ca1	2,081,797	0.000479
Affx_123249471	Ca1	2,083,627	0.000681
Affx_123277156	Ca6	41,065,459	0.000836
Affx_123286825	Ca1	2,123,224	0.000914
Affx_123247180	Ca1	39,608,692	0.000959
**Significant markers depicted by GLM showing association with *Helicoverpa armigera* larval population (2021)**			
Affx_123241676	Ca1	2,026,559	0.000107
Affx_123248246	Ca1	2,081,797	0.000114
Affx_123249471	Ca1	2,083,627	0.000148
Affx_123286825	Ca1	2,123,224	0.000207
Affx_123249101	Ca1	2,139,423	0.000328
Affx_123255526	Ca2	32,467,962	0.000389
Affx_123245103	Ca1	2,011,348	0.000469
Affx_123286346	Ca1	2,132,822	0.000491
Affx_123287807	Ca1	1,975,258	0.00061
Affx_123253041	Ca1	2,102,245	0.000691
Affx_123277156	Ca6	41,065,459	0.000709
Affx_123276567	Ca1	1,994,650	0.000787
Affx_123264792	Ca1	2,110,335	0.000794
Affx_123260724	Ca1	2,760,441	0.000795
**Significant markers depicted by MLM showing association with *Helicoverpa armigera* larval population (2021)**			
Affx_123266834	Ca6	43,044,918	0.000414
Affx_123255526	Ca2	32,467,962	0.000474
Affx_123241676	Ca1	2,026,559	0.000634
Affx_123242495	Ca1	35,358,570	0.000635
Affx_123248246	Ca1	2,081,797	0.000706
**Significant markers depicted by MLMM showing association with *Helicoverpa armigera* larval population (2021)**			
Affx_123266834	Ca6	43,044,918	0.000192
Affx_123255526	Ca2	32,467,962	0.000227
Affx_123241676	Ca1	2,026,559	0.000325
Affx_123242495	Ca1	35,358,570	0.000326
Affx_123248246	Ca1	2,081,797	0.000371
Affx_123249471	Ca1	2,083,627	0.00058
Affx_123277156	Ca6	41,065,459	0.000672
Affx_123286825	Ca1	2,123,224	0.000751
Affx_123247180	Ca1	39,608,692	0.000834
**Common SNPs found to be associated with *Helicoverpa armigera* larval population 2020 and 2021**			
Affx_123255526	Ca2	32,467,962	0.00022–0.00047
Affx_123241676	Ca1	2,026,559	0.00014–0.00066
Affx_123248246	Ca1	2,081,797	0.00017–0.0007

Abbreviations: GLM, general linear model; MLM, mixed linear model; MLMM, multi‐locus mixed model; SNP, single nucleotide polymorphism.

#### 3.2.2 GWAS signals for percentage pod damage by *H. armigera*

**FIGURE 3:**
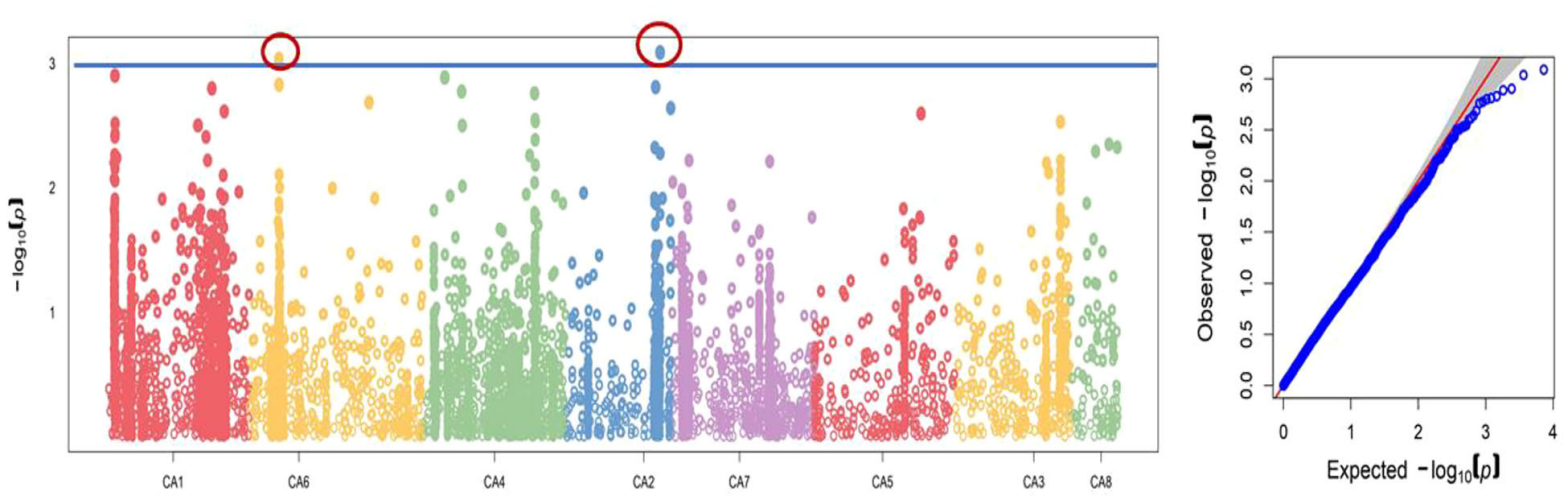
Manhattan plot and QQ plot showing candidate single nucleotide polymorphism (SNP) marker loci and *p*‐values from GWAS for *Helicoverpa armigera* resistance based on percent pod damage of year 2020 (The blue line on Manhattan plot is the fixed significance threshold of −log_10_ (*p* value) at 3).

**FIGURE 4:**
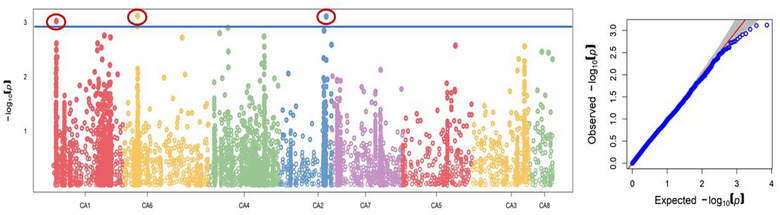
Manhattan plot and QQ plot showing candidate single nucleotide polymorphism (SNP) marker loci and *p*‐values from GWAS for *Helicoverpa armigera* resistance based on percent pod damage of year 2021 (The blue line on Manhattan plot is the fixed significance threshold of −log_10_ (*p* value) at 3).

Using GLM, we identified two significant MTAs (Affx_123249471 and Affx_123300587) for PPD based on 2021 phenotyping data on Ca1 and Ca4 (Table 3). However, using GLM, we did not detect any significant MTA based on 2020 data. While in case of PPD, no significant MTAs were identified using MLM during both year 2020 and 2021. Nevertheless, using MLMM, we identified two significant MTAs (Affx_123255526 and Affx_123279564), one each on Ca2 and Ca6 based on phenotyping data generated in 2020. Similarly, we identified three significant MTAs (Affx_123279564, Affx_123255526, and Affx_123249471) based on phenotyping data of 2021; however, one each was present on Ca6, Ca2, and Ca1 (Table 3). Interestingly, two common significant stable MTAs (Affx_123255526 and Affx_123279564) distributed on chromosomes Ca2 and Ca6 were found to be associated with PPD by *H. armigera* during both 2020 and 2021 (Table 3). A few representative Manhattan and QQ plots showing MTAs detected using different models are shown in Figures 3 and 4. The results indicate the usefulness of using MLMMs for GWAS since a single model has not been found to provide sufficient MTAs. The use of different models also assumed importance since we could identify markers that are discovered consistently in different models. The discovery of MTAs through different models could be useful and could be used in future chickpea breeding programmes aimed at enhancing chickpea pod borer resistance.

**TABLE 3:** Details of marker–trait associations (MTAs) identified for *Helicoverpa armigera* percentage pod damage using different GWAS (genome‐wide association studies) models in different years.

SNP	Chromosome	Position	*p* value
**Significant markers depicted by MLMM showing association with percentage pod damage of the year 2020**			
Affx_123255526	Ca2	32,467,962	0.000815
Affx_123279564	Ca6	10,137,098	0.000918
**Significant markers depicted by GLM showing association with percentage pod damage of the year 2021**			
Affx_123249471	Ca1	2,083,627	0.0008
Affx_123300587	Ca4	38,584,903	0.000993
**Significant markers depicted by MLMM showing association with percentage pod damage of the year 2021**			
Affx_123279564	Ca6	10,137,098	0.000758
Affx_123255526	Ca2	32,467,962	0.000777
Affx_123249471	Ca1	2,083,627	0.000943
**Common SNPs found to be associated with *Helicoverpa armigera* percentage pod damage 2020 and 2021**			
Affx_123255526	Ca2	32,467,962	0.00077–0.00081
Affx_123279564	Ca6	10,137,098	0.00075–0.00091

Abbreviations: GLM, general linear model; MLM, mixed linear model; MLMM, multi‐locus mixed model; SNP, single nucleotide polymorphism.

#### 3.2.3 GWAS signals for pest resistance

**FIGURE 5:**
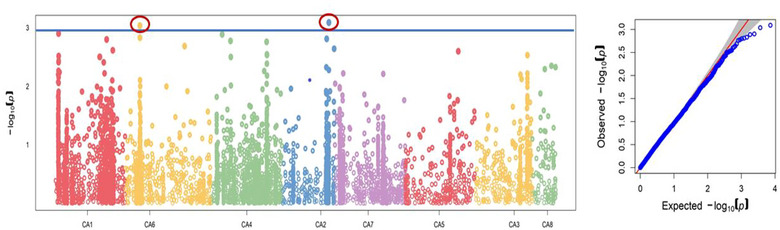
Manhattan plot and QQ plot showing candidate single nucleotide polymorphism (SNP) marker loci and *p*‐values from GWAS for *Helicoverpa armigera* pest resistance during year 2020 (The blue line on Manhattan plot is the fixed significance threshold of −log_10_ (*p* value) at 3).

**FIGURE 6:**
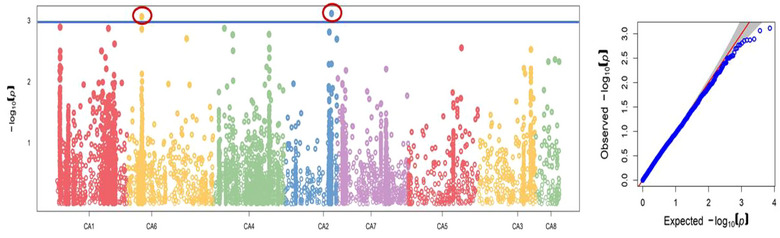
Manhattan plot and QQ plot showing candidate single nucleotide polymorphism (SNP) marker loci and *p*‐values from GWAS for *Helicoverpa armigera* pest resistance during the year 2021 (The blue line on Manhattan plot is the fixed significance threshold of −log_10_ (*p* value) at 3).

For pest resistance, no significant association was found during 2020 and 2021 using the GLM and MLMs, whereas the MLMM led to the identification of two SNPs (Affx_123255526 and Affx_123279564) associated with pest resistance during 2020, and these SNPs were distributed on chromosomes Ca2 and Ca6 (Table 4; Figure 5). Similarly, the MLMM also identified two SNPs associated with pest resistance during 2021, and these SNPs were distributed on chromosomes Ca2 and Ca6 (Table 4; Figure 6). These MTAs could potentially be used in breeding programs to develop chickpea varieties with enhanced resistance to pod borer infestation.

**TABLE 4:** Details of marker–trait associations (MTAs) identified for pest resistance using different GWAS (genome‐wide association studies) models in different years.

SNP	Chromosome	Position	*p* value
**Significant markers depicted by MLMM showing association with pest resistance of the year 2020**			
Affx_123255526	Ca2	32,467,962	0.000814
Affx_123279564	Ca6	10,137,098	0.000918
**Significant markers depicted by MLMM showing association with pest resistance of the year 2021**			
Affx_123255526	Ca2	32,467,962	0.000768
Affx_123279564	Ca6	10,137,098	0.000863
**Common SNPs found to be associated with pest resistance of the years 2020 and 2021**			
Affx_123255526	Ca2	32,467,962	0.000791
Affx_123279564	Ca6	10,137,098	0.00089
**Common SNP found to be associated with all the three (larval population, pod damage and pest resistance) component traits of *Helicoverpa armigera resistance***			
Affx_123255526	Ca2	32,467,962	0.00012–0.00081

Abbreviations: MLMM, multi‐locus mixed model; SNP, single nucleotide polymorphism.

The identification of MTAs for larval population, PPD, and overall pest resistance in chickpea against the notorious *H. armigera* represents a significant step forward in chickpea breeding programs aimed at enhancing resistance against this destructive pest. One of the MTAs (Affx_123255526) identified with all the three component traits of pod borer resistance (MLP, PPD, and overall pest resistance) could prove useful for chickpea breeding programs. In an earlier study reporting genes/QTLs for pod borer resistance in chickpea (Barmukh et al., 2021), several QTLs including main‐effect QTLs and epistatic QTLs associated with various aspects of *H. armigera* resistance were identified. The identification of multiple MTAs during the present study again highlights quantitative nature of pod borer resistance in chickpea. We identified MTAs on chromosomes Ca1, Ca2, Ca4, and Ca6, while Barmukh et al. (2021) reported QTLs on chromosomes Ca3 and Ca4. Therefore, genes/QTLs have been reported on some new chromosomes by us during the present study may be unique and important for chickpea breeding. However, the position of these QTLs reporting on same chromosomes could not be compared since our study is GWAS and earlier study by Barmukh et al. (2021) is a QTL mapping study. By leveraging the knowledge gained from the present study and that of Barmukh et al. (2021), breeders can mitigate the economic losses caused by *H. armigera* infestations while reducing reliance on chemical pesticides, thus promoting sustainable chickpea production worldwide.

### 3.3 Candidate genes for insect resistance

Out of 17 unique SNP positions that were associated with larval population, only Affx_123241676, Affx_123248246, Affx_123276567, Affx_123264792, and Affx_123260724 were present within the exonic regions of chickpea genomic assembly—v1.0 (Varshney et al., 2013). The SNP, Affx_123241676, is present in the exonic region of gene Ca_00256 that codes for Secretory Carrier Associated Membrane Protein (SCAMP). SCAMP protein was reported to play a key role in plant defense participating in membrane trafficking processes that facilitate the secretion of defense‐related molecules. In the case of *Arabidopsis thaliana*, it was demonstrated that the SCAMPs are involved in the secretion of defensive proteins, such as protease inhibitors and toxins, which can hinder insect feeding or growth (Karnik et al., 2013). These defense proteins are often stored in vesicles and are released upon insect attack.

The SNP, Affx_123248246, present in Ca_03264 gene codes for FGGY carbohydrate kinases. FGGY carbohydrate kinases are known to participate in the phosphorylation of carbohydrates, including sugars, sugar alcohols, and sugar phosphates. This phosphorylation is often a critical step in the metabolism and utilization of carbohydrates. While the specific involvement of FGGY carbohydrate kinases in plant defense against pests requires further investigation, their participation in carbohydrate metabolism suggests a potential indirect role in plant immune responses. Another SNP, “Affx_123276567” present in a gene “Ca_00249” that encodes quinone oxidoreductase (QORs). The QORs is a class of enzymes that play diverse roles in cellular metabolism, including the metabolism of quinones and other compounds involved in oxidative reactions and production of reactive oxygen species (ROS) during the oxidative burst, which is an early defense response. ROS, such as hydrogen peroxide and superoxide, are generated when plants experience biotic and abiotic stress, which leads to oxidative stress in plant that is lethal for plant growth. The identified Affx_123276567 SNP within the Ca_00249 gene underscores the crucial role of QORs in alleviating oxidative stress and countering ROS production during the oxidative burst induced by *Helicoverpa* infestation. Examining the genetic variations in this gene and its associated SNP offers valuable insights into the potential implications for the plant's response to oxidative stress and its defense mechanisms at the cellular level. Furthermore, supporting evidence from multiple reports, including the study by Dowd and Johnson (2023) and Heyno et al. (2013), indicates that QORs operate as flavano proteins, actively serving as a shield for plants against oxidative stress. This protective function is consistent with the recognized significance of QORs in preserving cellular homeostasis and reinforcing plant resilience, particularly in the face of biotic stress. Nevertheless, ROS were reported to promote diapause that extends life span in pupae of the moth *H. armigera* (Zhang et al., 2017). The SNP Affx_123264792 is present in a gene Ca_00267 that encodes for Katanin p80 WD40‐containing subunit B1. KATANIN B1 is a protein involved in microtubule severing, which is important for cytoskeleton remodeling and various cellular processes in plants. Plant defense against pathogens often involves dynamic changes in the cytoskeleton, including microtubule rearrangements, which are necessary for processes such as cell wall reinforcement, vesicle trafficking, and signaling cascades. The severing of microtubules by KATANIN B1 can contribute to cytoskeletal remodeling and may play a role in plant defense responses. Another significant MTA, Affx_123260724, present in the gene Ca_00340 codes for NB‐LRR (nucleotide‐binding domain and leucine‐rich repeat) type disease resistance protein RPS1‐K‐2 (also known as RPS1‐K or RPS1‐K2). NB‐LRR RPS1‐K‐2 is a specific member of the NB‐LRR protein family in plants. It is known to play a critical role in plant disease resistance against certain pathogens. The NB‐LRR type disease resistance protein RPS1‐K‐2 has not been specifically studied for its role in *Helicoverpa* disease resistance. However, other NB‐LRR proteins have been implicated in plant defense against *H. armigera*, a common insect pest known as the cotton bollworm or corn earworm (Walsh et al., 2018). Barmukh et al. (2021) reported that a QTL cluster on CaLG03 holds great potential to improve *H. armigera* resistance component traits in chickpea.

## 4 CONCLUSION

Gram pod borer is one of the major constraints of chickpea production. Several approaches have been utilized for managing this pest, but all have limited effectiveness. The key to manage this key pest is to understand and exploit the genetic resistance and develop more durable cultivars for the future. Genotypes NK 7, NK 13, NK59, NK 63, NK124, Y 14, NK 48, CC110, and NK 122 were found to be resistant during both the years, namely, year 2020 and year 2021.Thus, these genotypes can be considered as validated for their resistance against the pod borer. The trait data on pod borer resistance and genotypic data of *Axiom CicerSNP Array* were used together to identify 53 significant MTAs. A set of three MTAs was found to be common in both environments (years 2020 and 2021) and are therefore very important for chickpea breeding programs. Similarly, the analysis of MTAs for PPD using different models led to the identification of two MTAs in year 2020 and five MTAs in year 2021, and a set of two MTAs were common in both environments (2020 and 2021). In addition, we reported key genes that encode SCAMPs, QORs, and NB‐LRR proteins and have been implicated in plant defense against *H*. *armigera*. The resistant genotypes, MTAs, and key genes reported in the present study may be deployed for developing pod borer–resistant chickpea varieties.

## AUTHOR CONTRIBUTIONS

**Sheikh Aafreen Rehman**: Formal analysis; investigation; methodology; software; writing—original draft; writing—review and editing. **Shaheen Gul**: Conceptualization; supervision; writing—original draft. **M. Parthiban**: Data curation; formal analysis; software; validation; writing—review and editing. **Ishita Isha**: Validation; writing—review and editing. **M. S. Sai Reddy**: Formal analysis; validation. **Annapurna Chitikineni**: Data curation; formal analysis; funding acquisition; writing—original draft. **Mahendar Thudi**: Conceptualization; funding acquisition; project administration; software; writing—original draft; writing—review and editing. **R. Varma Penmetsa**: Conceptualization; formal analysis; supervision; writing—review and editing. **Rajeev Kumar Varshney**: Conceptualization; data curation; formal analysis; funding acquisition; investigation; project administration; resources; software; supervision; validation; writing—original draft; writing—review and editing. **Reyazul Rouf Mir**: Conceptualization; funding acquisition; project administration; resources; supervision; writing—original draft; writing—review and editing.

## CONFLICT OF INTEREST STATEMENT

The authors declare no conflicts of interest.

## Supporting information

Supplementary table 1: Pest resistance (%age) scale used for recording data on pest resistance/pod borer damage.
Supplementary table 2: Germplasm evaluation under field conditions during year 2020 and year 2021. The table shows data recorded on Mean larval population (MLP), Percent pod damage (PPD), and Pest resistance (PR).

## Data Availability

All the data are available in the manuscript.
