# Supplementary material for: Genetic resources and genes/QTLs for gram pod borer (Helicoverpa armigera Hübner) resistance in chickpea from the Western Himalayas
Source: Plant Genome. 2024 Jul 4;17(3):e20483. doi: 10.1002/tpg2.20483 (PMC12807025; doi:10.1002/tpg2.20483)
Supplement: Supplementary file 1 [file TPG2-17-e20483-s001.docx]

| **Supplementary table 1: Pest resistance (%age) scale used for recording data on pest resistance/pod borer damage.** | | |
| --- | --- | --- |
| **Pest Resistance** | **Grade** | **Category** |
| 100% | 1 | Immune (IM) |
| 75 to 99% | 2 | Highly Resistant (HR) |
| 50 to 75% | 3 | Resistant (R) |
| 25 to 50% | 4 | Moderately Resistant (MR) |
| 0 to 25% | 5 | Intermediate(I) |
| -25 to 0 | 6 | Moderately Susceptible (MS) |
| -50 to -25% | 7 | Susceptible (S) |
| <-50% | 8 | Highly Susceptible (HS) |

| **Supplementary table 2: Germplasm evaluation under field conditions during year 2020 and year 2021. The table shows data recorded on Mean larval population (MLP), Percent pod damage (PPD), and Pest resistance (PR).** | | | | | | | | | |
| --- | --- | --- | --- | --- | --- | --- | --- | --- | --- |
| S. No. | Genotype | MLP  (2020) | MLP  (2021) | PPD  (2020) | PPD  (2021) | PR (2020) | PR  (2021) | Category  (2020) | Category  (2021) |
| 1 | CC 11 | 0.72 | 0.85 | 26.02 | 27.35 | -280.40 | -269.59 | HS | HS |
| 2 | CC 12 | 0.45 | 0.75 | 9.25 | 9.35 | -35.18 | -26.35 | S | S |
| 3 | CC 14 | 0.69 | 0.73 | 18.35 | 19.49 | -168.27 | -163.37 | HS | HS |
| 4 | CC 16 | 0.38 | 0.42 | 8.18 | 8.21 | -19.63 | -10.94 | MS | MS |
| 5 | CC 19 | 0.37 | 0.41 | 7.89 | 8.01 | -15.39 | -8.24 | MS | MS |
| 6 | CC 27 | 0.44 | 0.44 | 9.64 | 9.74 | -40.88 | -31.62 | S | S |
| 7 | CC 29 | 0.34 | 0.38 | 6.98 | 7.44 | -2.046 | -0.54 | MS | MS |
| 8 | CC 31 | 2.08 | 2.13 | 33.28 | 33.95 | -386.59 | -358.78 | HS | HS |
| 9 | CC32 | 0.44 | 0.45 | 12.06 | 12.25 | -76.36 | -65.54 | HS | HS |
| 10 | CC 34 | 0.34 | 0.39 | 8.35 | 8.40 | -22.02 | -13.51 | MS | MS |
| 11 | CC 35 | 0.40 | 0.38 | 7.02 | 7.03 | -2.58 | 5 | MS | I |
| 12 | CC 38 | 3.27 | 0.41 | 8.81 | 8.91 | -28.80 | -20.40 | S | MS |
| 13 | CC 39 | 0.36 | 3.28 | 45.58 | 45.68 | -566.32 | -517.29 | HS | HS |
| 14 | CC 41 | 3.53 | 0.40 | 7.62 | 7.68 | -11.40 | -3.78 | MS | MS |
| 15 | CC43 | 0.68 | 3.57 | 53.96 | 54.11 | -688.93 | -631.21 | HS | HS |
| 16 | CC 48 | 0.72 | 0.71 | 16.64 | 16.65 | -143.27 | -125 | HS | HS |
| 17 | CC49 | 0.64 | 0.77 | 20.92 | 20.96 | -205.84 | -183.24 | HS | HS |
| 18 | CC50 | 1.09 | 0.68 | 14.49 | 14.68 | -111.79 | -98.37 | HS | HS |
| 19 | CC52 | 3.83 | 1.10 | 29.86 | 29.88 | -336.59 | -303.78 | HS | HS |
| 20 | CC57 | 0.34 | 4.09 | 59.24 | 59.60 | -766.08 | -705.40 | HS | HS |
| 21 | CC 61 | 0.79 | 0.80 | 25.53 | 25.99 | -273.29 | -251.21 | HS | HS |
| 22 | CC63 | 0.34 | 0.38 | 6.63 | 6.76 | 3.021 | 8.64 | I | I |
| 23 | CC 65 | 0.91 | 0.89 | 25.86 | 26.03 | -278.11 | -251.75 | HS | HS |
| 24 | CC 74 | 0.53 | 0.56 | 10.02 | 10.08 | -46.49 | -36.21 | S | S |
| 25 | CC 76 | 0.30 | 0.33 | 5.43 | 5.46 | 20.56 | 26.21 | I | MR |
| 26 | CC 83 | 0.67 | 0.71 | 19.43 | 19.47 | -184.06 | -163.10 | HS | HS |
| 27 | CC 87 | 0.65 | 0.68 | 17.87 | 17.91 | -161.20 | -142.02 | HS | HS |
| 28 | CC 88 | 0.40 | 0.45 | 8.42 | 8.45 | -23.05 | -14.18 | MS | MS |
| 29 | CC 92 | 0.29 | 0.32 | 6.03 | 6.03 | 11.84 | 18.51 | I | I |
| 30 | CC 96 | 0.40 | 0.42 | 8.73 | 8.77 | -27.63 | -18.51 | S | MS |
| 31 | CC 98 | 0.61 | 0.65 | 13.70 | 13.77 | -100.34 | -86.081 | HS | HS |
| 32 | CC 102 | 2.45 | 2.47 | 35.86 | 36.00 | -424.31 | -386.48 | HS | HS |
| 33 | CC 107 | 0.35 | 0.37 | 6.96 | 7.00 | -1.8031 | 5.40 | MS | I |
| 34 | CC 111 | 3.00 | 3.04 | 40.33 | 40.64 | -489.66 | -449.18 | HS | HS |
| 35 | CC 113 | 0.41 | 0.45 | 8.95 | 8.95 | -30.79 | -20.94 | S | MS |
| 36 | CC 114 | 0.61 | 0.64 | 10.50 | 11.74 | -53.55 | -58.64 | HS | HS |
| 37 | CC 117 | 0.60 | 0.63 | 13.57 | 13.56 | -98.44 | -83.24 | HS | HS |
| 38 | CC 118 | 0.52 | 0.53 | 10.04 | 10.06 | -46.73 | -35.94 | S | S |
| 39 | CC 120 | 4.15 | 4.16 | 60.45 | 61.10 | -783.77 | -725.67 | HS | HS |
| 40 | CC 124 | 0.24 | 0.26 | 4.12 | 4.29 | 39.71 | 42.027 | MR | MR |
| 41 | CC 126 | 0.71 | 0.75 | 19.42 | 19.44 | -183.91 | -162.70 | HS | HS |
| 42 | CC 130 | 0.35 | 0.39 | 7.40 | 7.42 | -8.13 | -0.27 | MS | MS |
| 43 | CC 131 | 0.35 | 0.37 | 6.67 | 6.67 | 2.48 | 9.86 | I | I |
| 44 | CC 133 | 0.45 | 0.48 | 12.14 | 12.15 | -77.43 | -64.18 | HS | HS |
| 45 | CC 140 | 0.61 | 0.66 | 10.22 | 10.28 | -49.36 | -38.91 | S | S |
| 46 | CC 144 | 0.40 | 0.44 | 8.78 | 8.79 | -28.41 | -18.78 | S | MS |
| 47 | CC 145 | 0.62 | 0.72 | 10.75 | 10.82 | -57.11 | -46.21 | HS | S |
| 48 | CC 146 | 0.41 | 0.48 | 11.95 | 12.00 | -74.65 | -62.16 | HS | HS |
| 49 | CC 147 | 0.37 | 0.44 | 8.28 | 8.35 | -21.10 | -12.83 | MS | MS |
| 50 | CC 150 | 0.29 | 0.29 | 5.88 | 5.88 | 14.08 | 20.54 | I | I |
| 51 | CC 151 | 0.67 | 0.75 | 17.09 | 17.89 | -149.90 | -141.75 | HS | HS |
| 52 | CC 152 | 0.68 | 0.72 | 17.25 | 17.37 | -152.19 | -134.73 | HS | HS |
| 53 | CC 154 | 0.31 | 0.32 | 6.01 | 6.08 | 12.183 | 17.83 | I | I |
| 54 | CC 162 | 0.67 | 0.69 | 14.60 | 14.66 | -113.40 | -98.10 | HS | HS |
| 55 | CC 168 | 0.26 | 0.29 | 4.31 | 4.31 | 36.98 | 41.75 | MR | MR |
| 56 | CC 169 | 0.86 | 0.32 | 25.12 | 25.20 | -267.25 | -240.54 | HS | HS |
| 57 | CC 171 | 0.65 | 0.69 | 15.83 | 15.88 | -131.38 | -114.59 | HS | HS |
| 58 | CC 175 | 0.61 | 0.64 | 14.32 | 14.40 | -109.40 | -94.59 | HS | HS |
| 59 | CC 179 | 0.47 | 0.52 | 12.40 | 12.42 | -81.33 | -67.83 | HS | HS |
| 60 | CC 184 | 0.85 | 0.88 | 27.09 | 27.22 | -296.10 | -267.83 | HS | HS |
| 61 | CC 187 | 1.33 | 1.33 | 30.50 | 30.59 | -345.95 | -313.37 | HS | HS |
| 62 | CC 188 | 0.33 | 0.36 | 6.41 | 6.53 | 6.23 | 11.75 | I | I |
| 63 | CC 189 | 0.46 | 0.46 | 9.55 | 9.75 | -39.57 | -31.75 | S | S |
| 64 | CC 191 | 0.71 | 0.71 | 17.54 | 17.59 | -156.43 | -137.70 | HS | HS |
| 65 | CC 193 | 0.68 | 0.69 | 14.82 | 14.94 | -116.66 | -101.89 | HS | HS |
| 66 | CC 196 | 0.77 | 0.81 | 21.43 | 21.53 | -213.25 | -190.94 | HS | HS |
| 67 | CC 198 | 0.31 | 0.77 | 6.73 | 6.79 | 1.55 | 8.24 | I | I |
| 68 | CC 199 | 0.33 | 0.37 | 6.71 | 6.73 | 1.85 | 9.05 | I | I |
| 69 | CP 2 | 0.34 | 0.37 | 6.25 | 6.30 | 8.67 | 14.86 | I | I |
| 70 | CP 3 | 0.33 | 0.36 | 5.82 | 5.96 | 14.96 | 19.45 | I | I |
| 71 | CP 4 | 0.62 | 0.65 | 10.75 | 10.98 | -57.11 | -48.37 | HS | S |
| 72 | CP 6 | 0.64 | 0.65 | 14.50 | 14.76 | -111.94 | -99.45 | HS | HS |
| 73 | CP 9 | 0.36 | 0.40 | 6.72 | 6.74 | 1.70 | 8.91 | I | I |
| 74 | CP 11 | 0.71 | 0.77 | 20.43 | 20.49 | -198.63 | -176.89 | HS | HS |
| 75 | CP 12 | 0.69 | 0.73 | 10.50 | 11.66 | -53.46 | -57.56 | HS | HS |
| 76 | CP 13 | 0.41 | 0.48 | 9.30 | 9.39 | -35.91 | -26.89 | S | S |
| 77 | CP 14 | 0.71 | 0.73 | 19.02 | 19.73 | -178.07 | -166.62 | HS | HS |
| 78 | CP 15 | 0.33 | 0.38 | 6.46 | 6.46 | 5.55 | 12.70 | I | I |
| 79 | CP 16 | 0.69 | 0.73 | 14.74 | 14.74 | -115.49 | -99.18 | HS | HS |
| 80 | CP 19 | 0.59 | 0.63 | 13.18 | 13.24 | -92.64 | -78.91 | HS | HS |
| 81 | CP 20 | 0.65 | 0.69 | 15.76 | 15.80 | -130.40 | -113.51 | HS | HS |
| 82 | CP 21 | 0.79 | 0.83 | 22.39 | 22.55 | -227.29 | -204.73 | HS | HS |
| 83 | CP 28 | 0.57 | 0.60 | 13.04 | 13.07 | -90.69 | -76.62 | HS | HS |
| 84 | CP 29 | 0.85 | 0.81 | 25.82 | 25.86 | -277.48 | -249.45 | HS | HS |
| 85 | CP 30 | 0.36 | 0.40 | 7.61 | 7.89 | -11.30 | -6.62 | MS | MS |
| 86 | NK 1 | 0.47 | 0.51 | 12.23 | 12.34 | -78.80 | -66.75 | HS | HS |
| 87 | NK 2 | 0.41 | 0.44 | 9.96 | 10.01 | -45.66 | -35.27 | S | S |
| 88 | NK 3 | 0.22 | 0.26 | 3.44 | 3.90 | 49.75 | 47.29 | MR | MR |
| 89 | NK 4 | 0.33 | 0.38 | 2.54 | 6.90 | 62.91 | 6.75 | R | I |
| 90 | NK 7 | 0.20 | 0.25 | 2.50 | 2.54 | 63.45 | 65.67 | R | R |
| 91 | NK 9 | 0.68 | 0.71 | 11.20 | 11.26 | -63.79 | -52.16 | HS | HS |
| 92 | NK 10 | 0.61 | 0.68 | 14.32 | 14.39 | -109.30 | -94.45 | HS | HS |
| 93 | NK 11 | 0.60 | 0.64 | 13.68 | 13.78 | -100 | -86.21 | HS | HS |
| 94 | NK 12 | 0.59 | 0.64 | 12.22 | 12.53 | -78.60 | -69.32 | HS | HS |
| 95 | NK 13 | 0.21 | 0.23 | 2.56 | 2.84 | 62.57 | 61.62 | R | R |
| 96 | NK 22 | 0.37 | 0.41 | 8.05 | 8.15 | -17.64 | -10.13 | MS | MS |
| 97 | NK 23 | 0.29 | 0.33 | 5.13 | 5.17 | 25 | 30.13 | MR | MR |
| 98 | NK 25 | 0.64 | 0.67 | 17.78 | 17.87 | -159.94 | -141.48 | HS | HS |
| 99 | NK 31 | 0.28 | 0.33 | 4.78 | 5.04 | 30.16 | 31.89 | MR | MR |
| 100 | NK 59 | 0.21 | 0.28 | 3.07 | 3.17 | 55.06 | 57.16 | R | R |
| 101 | NK 63 | 0.20 | 0.25 | 2.50 | 2.57 | 63.49 | 65.27 | R | R |
| 102 | NK 65 | 0.67 | 0.72 | 25.49 | 25.54 | -272.61 | -245.13 | HS | HS |
| 103 | NK 70 | 0.26 | 0.32 | 4.30 | 4.61 | 37.08 | 37.70 | MR | MR |
| 104 | NK 75 | 0.28 | 0.32 | 4.63 | 4.94 | 32.30 | 33.24 | MR | MR |
| 105 | NK 88 | 0.40 | 0.40 | 9.25 | 9.55 | -35.28 | -29.05 | S | S |
| 106 | NK 114 | 0.33 | 0.41 | 7.28 | 7.55 | -6.38 | -2.02 | MS | MS |
| 107 | NK 116 | 0.35 | 0.40 | 36.04 | 36.51 | -426.94 | -393.37 | HS | HS |
| 108 | NK 124 | 0.22 | 0.26 | 2.67 | 2.82 | 61.01 | 61.89 | R | R |
| 109 | NK 35 | 0.69 | 0.72 | 16.87 | 17.07 | -146.68 | -130.67 | HS | HS |
| 110 | Y 1 | 0.25 | 0.30 | 4.19 | 4.89 | 38.79 | 33.91 | MR | MR |
| 111 | Y2 | 2.60 | 2.64 | 38.03 | 38.11 | -456.04 | -415 | HS | HS |
| 112 | Y3 | 0.68 | 0.72 | 11.47 | 11.73 | -67.69 | -58.51 | HS | HS |
| 113 | Y4 | 0.59 | 0.63 | 13.10 | 13.40 | -91.47 | -81.08 | HS | HS |
| 114 | Y 7 | 0.28 | 0.33 | 4.75 | 4.96 | 30.50 | 32.97 | MR | MR |
| 115 | Y 11 | 0.36 | 0.44 | 7.63 | 7.78 | -11.50 | -5.13 | MS | MS |
| 116 | Y 12 | 5.01 | 5.03 | 67.90 | 68.80 | -892.73 | -829.73 | HS | HS |
| 117 | Y 14 | 0.21 | 0.24 | 3.38 | 3.65 | 50.53 | 50.67 | R | R |
| 118 | Y 17 | 0.41 | 0.42 | 46.79 | 46.80 | -584.06 | -532.43 | HS | HS |
| 119 | NK 48 | 0.20 | 0.22 | 3.27 | 3.49 | 52.14 | 52.83 | R | R |
| 120 | Y8 | 0.40 | 0.41 | 11.78 | 11.83 | -72.22 | -59.86 | HS | HS |
| 121 | NK 55 | 0.24 | 0.26 | 3.98 | 4.01 | 41.86 | 45.81 | MR | MR |
| 122 | NK 41 | 0.38 | 0.41 | 8.17 | 9.00 | -19.49 | -21.62 | MS | MS |
| 123 | K 35 | 0.59 | 0.59 | 13.17 | 13.23 | -92.49 | -78.78 | HS | HS |
| 124 | K 40 | 0.47 | 0.49 | 12.44 | 12.53 | -81.87 | -69.32 | HS | HS |
| 125 | CP 27 | 0.67 | 0.69 | 8.31 | 8.35 | -21.49 | -12.83 | MS | MS |
| 126 | CP 25 | 0.26 | 0.30 | 4.31 | 4.50 | 36.98 | 39.18 | MR | MR |
| 127 | H1 | 0.63 | 0.67 | 13.10 | 13.20 | -91.52 | -78.37 | HS | HS |
| 128 | H4 | 0.26 | 0.30 | 4.01 | 4.03 | 41.32 | 45.54 | MR | MR |
| 129 | H5 | 0.36 | 0.39 | 7.55 | 7.63 | -10.38 | -3.10 | MS | MS |
| 130 | H 8 | 0.41 | 0.44 | 8.43 | 8.48 | -23.19 | -14.59 | MS | MS |
| 131 | H 19 | 0.67 | 0.71 | 11.10 | 11.13 | -62.28 | -50.40 | HS | HS |
| 132 | H 9 | 0.81 | 0.81 | 23.12 | 23.12 | -238.06 | -212.4 | HS | HS |
| 133 | H 3 | 5.45 | 5.47 | 73.39 | 74.78 | -972.90 | -910.54 | HS | HS |
| 134 | K 32 | 0.47 | 0.49 | 12.23 | 12.44 | -78.75 | -68.10 | HS | HS |
| 135 | H 13 | 0.33 | 0.39 | 6.95 | 7.07 | -1.65 | 4.45 | MS | I |
| 136 | CP 22 | 0.40 | 0.42 | 11.96 | 12.02 | -74.80 | -62.43 | HS | HS |
| 137 | H 14 | 0.49 | 0.53 | 12.83 | 12.90 | -87.62 | -74.32 | HS | HS |
| 138 | H 18 | 0.32 | 0.36 | 5.64 | 5.75 | 17.49 | 22.29 | I | I |
| 139 | K 7 | 0.35 | 0.40 | 6.96 | 6.98 | -1.75 | 5.67 | MS | I |
| 140 | H 12 | 0.61 | 0.64 | 13.27 | 13.39 | -93.95 | -80.94 | HS | HS |
| 141 | CC 60 | 0.68 | 0.71 | 16.53 | 17.15 | -141.66 | -131.75 | HS | HS |
| 142 | CC 64 | 0.61 | 0.62 | 10.74 | 10.98 | -57.01 | -48.37 | HS | S |
| 143 | CC 99 | 0.32 | 0.34 | 5.64 | 6.53 | 17.54 | 11.75 | I | I |
| 144 | CC 110 | 0.25 | 0.29 | 2.86 | 2.95 | 58.18 | 60.13 | R | R |
| 145 | CC 185 | 0.71 | 0.72 | 19.02 | 19.10 | -178.11 | -158.10 | HS | HS |
| 146 | CP 27 | 0.69 | 0.71 | 11.08 | 11.42 | -61.93 | -54.32 | HS | HS |
| 147 | NK 14 | 0.71 | 0.75 | 20.37 | 20.43 | -197.75 | -176.08 | HS | HS |
| 148 | NK 32 | 0.34 | 0.34 | 9.90 | 10.02 | -44.73 | -35.40 | S | S |
| 149 | NK 38 | 0.31 | 3.07 | 9.72 | 9.80 | -42.15 | -32.43 | S | S |
| 150 | NK 51 | 0.33 | 0.38 | 6.46 | 6.53 | 5.60 | 11.75 | I | I |
| 151 | NK 68 | 0.68 | 0.72 | 16.53 | 16.67 | -141.66 | -125.27 | HS | HS |
| 152 | NK 72 | 0.84 | 0.87 | 23.94 | 24.04 | -250 | -224.86 | HS | HS |
| 153 | NK 74 | 0.71 | 0.73 | 19.02 | 19.25 | -178.07 | -160.13 | HS | HS |
| 154 | NK 78 | 0.60 | 0.61 | 13.66 | 13.73 | -99.70 | -85.54 | HS | HS |
| 155 | NK 89 | 0.67 | 0.71 | 15.94 | 16.16 | -133.09 | -118.37 | HS | HS |
| 156 | NK 103 | 0.35 | 0.39 | 6.82 | 10.35 | 0.29 | -39.86 | I | S |
| 157 | NK 104 | 0.67 | 0.71 | 16.31 | 16.47 | -138.40 | -122.56 | HS | HS |
| 158 | NK 106 | 0.32 | 0.37 | 7.14 | 7.17 | -4.33 | 3.10 | MS | I |
| 159 | NK 107 | 0.42 | 0.45 | 9.72 | 9.79 | -42.15 | -32.29 | S | S |
| 160 | NK 112 | 0.63 | 0.65 | 14.43 | 14.50 | -110.96 | -95.94 | HS | HS |
| 161 | NK 118 | 0.60 | 0.62 | 10.21 | 10.31 | -49.22 | -39.32 | S | S |
| 162 | NK 122 | 0.22 | 0.24 | 2.70 | 2.70 | 60.52 | 63.51 | R | R |
| 163 | NK 129 | 0.64 | 0.68 | 10.75 | 10.75 | -57.11 | -45.27 | HS | S |
| 164 | NK 130 | 0.36 | 0.41 | 7.11 | 7.15 | -3.94 | 3.37 | MS | I |
| 165 | NK 144 | 0.30 | 0.34 | 4.69 | 4.71 | 31.43 | 36.35 | MR | MR |
| 166 | NK 147 | 0.48 | 0.51 | 12.37 | 12.40 | -80.79 | -67.56 | HS | HS |
| 167 | NK 148 | 0.37 | 0.40 | 9.00 | 9.00 | -31.57 | -21.62 | S | MS |
| 168 | NK 150 | 0.60 | 0.65 | 14.28 | 14.31 | -108.77 | -93.37 | HS | HS |
| 169 | H 10 | 0.67 | 0.62 | 8.33 | 8.38 | -21.78 | -13.24 | MS | MS |
| 170 | H22 | 0.61 | 0.64 | 12.54 | 12.60 | -83.33 | -70.27 | HS | HS |
| 171 | Y 10 | 0.35 | 0.37 | 6.84 | 7.4 | - | - | - | - |
| 172 | Y 13 | 0.82 | 0.83 | 25.64 | 26.01 | - | - | - | - |
| 173 | Y 5 | 0.68 | 0.70 | 20.26 | 20.5 | - | - | - | - |
| MLP (2020): Mean larval population during the year 2020  MLP (2021): Mean larval population during the year 2021  PPD (2020): Percent pod damage during the year 2020  PPD (2021): Percent pod damage during the year 2021  PR (2020): Pest resistance during the year 2020  PR (2021): Pest resistance during the year 2021 | | | | | | | | | |
